# Intra-Amniotic Inflammatory Response in Subgroups of Women with Preterm Prelabor Rupture of the Membranes

**DOI:** 10.1371/journal.pone.0043677

**Published:** 2012-08-20

**Authors:** Teresa Cobo, Marian Kacerovsky, Montse Palacio, Helena Hornychova, David M. Hougaard, Kristin Skogstrand, Bo Jacobsson

**Affiliations:** 1 Maternal-Fetal Medicine Department, ICGON, Hospital Clinic, Universitat de Barcelona, Barcelona, Spain; 2 Fetal and Perinatal Medicine Research Group, Institut d'Investigacions Biomediques August Pi i Sunyer (IDIBAPS), Barcelona, Spain; 3 Centro de Investigación Biomédica en Red de Enfermedades Raras (CIBERER), Barcelona, Spain; 4 Biomedical Research Center, University Hospital Hradec Kralove, Department of Obstetrics and Gynecology, Charles University in Praque, Faculty Medicine Hradec Kralove, Hradec Kralove, Czech Republic; 5 Fingerland's Department of Pathology, University Hospital Hradec Kralove, Hradec Kralove, Czech Republic; 6 Department of Clinical Biochemistry and Immunology, Statens Serum Institut, Copenhagen, Denmark; 7 Department of Obstetrics and Gynecology, Sahlgrenska University Hospital, Gothenburg, Sweden; 8 Institute of Public Health, Oslo, Norway; Institut Jacques Monod – UMR 7592 CNRS – Université Paris Diderot, France

## Abstract

**Background:**

To evaluate the influence of microbial invasion of the amniotic cavity (MIAC) and histological chorioamnionitis (HCA) on the magnitude of intra-amniotic inflammatory response in preterm prelabor rupture of membranes (PPROM).

**Methodology/Principal Finding:**

A prospective cohort study was performed in 107 women with PPROM between 23.0 and 36.6 weeks of gestational age. Twenty-six proteins were assayed by multiple immunoassay in amniotic fluid. The policy for PPROM in Czech Republic is active, and 90% of the women were delivered within 96 hours of membrane rupture. Histopathological placental findings were evaluated based on the Salafia classification. Data were analyzed in four subgroups of population according to the presence of MIAC and/or HCA. Results were stratified by gestational age at PPROM (< or ≥34.0 weeks). The rates of MIAC and HCA were 44% and 57%, respectively. Regardless of gestational age at PPROM, intra-amniotic inflammatory response was higher when MIAC and HCA were both present. There were no differences in the intra-amniotic inflammatory response between women with MIAC or HCA alone and women without infection.

**Conclusion:**

A higher intra-amniotic inflammatory response was identified when both HCA and MIAC were detected.

## Introduction

Microbial invasion of the amniotic cavity (MIAC) has been extensively related to neonatal morbidity and mortality in pregnancies with preterm prelabor rupture of membranes (PPROM) [1]–[5]. However, the presence of intra-amniotic inflammatory status seems to be as important for the occurrence of preterm delivery and neonatal complications as the identification of microorganisms in the amniotic fluid [6]–[11]. Higher intra-amniotic inflammatory response has been reported in the presence of MIAC [6]–[9] and in the presence of histological chorioamnionitis (HCA) [10], [11], suggesting that not only the amniotic cavity but also the placenta may play a role in the inflammatory response.

Few studies have explored the intensity of the intra-amniotic inflammatory response in different compartments (e.g. amniotic fluid and placenta or different parts of the placenta and the fetus). In a mixed group of women with HCA who delivered (spontaneously and electively) before 36 weeks, Parker et al [12] observed a more intense intra-amniotic inflammatory response and a worse neonatal outcome when the inflammatory process involved the amnion layer in addition to the chorion layer, suggesting that the deeper the placental involvement the more severe the inflammatory response. Davies et al [13] evaluated the natural course of acute inflammation in the placenta, uterus and fetal lung in a rabbit model infected by *Escherichia coli* and reported earlier response to inflammation in the uterine tissue and the placenta than in the fetal lung.

The policy of active management of women with PPROM in the Czech Republic [14] provides a unique possibility for evaluation of the amniotic fluid, placenta and umbilical cord with little time discrepancies between sampling of the different compartments.

Since intra-amniotic inflammation seems to be a risk factor for morbidity in PPROM at least as important as MIAC, the aim of this study was to evaluate the magnitude of intra-amniotic inflammatory response according to the presence of MIAC and HCA.

## Methods

### Study population and procedures

We studied pregnant women between 23.0 to 36.6 weeks of gestation with a diagnosis of PPROM who were admitted to the Department of Obstetrics and Gynecology, University Hospital Hradec Kralove, Czech Republic, between July 2008 and October 2010. Gestational age was established according to the first-trimester ultrasound scan. Multiple pregnancies, structural/chromosomal anomalies and patients with clinical signs of chorioamnionitis or vaginal bleeding at admission were not considered eligible for this study.

PPROM was defined as leakage of amniotic fluid preceding the presence of cervical changes and the onset of uterine contractions. PPROM was diagnosed by a sterile speculum examination to identify pooling of amniotic fluid in the vagina in association with a positive test for the presence of insulin-like growth factor–binding protein (ACTIM PROM test; MedixBiochemica, Kauniainen, Finland) in the vaginal fluid.

A complete course of antenatal steroids, betamethasone 12-mg intramuscular injection with two doses given 24 h apart, was administered when PPROM occurred between 24.0 to 33.6 weeks. Tocolysis was considered for 48 h in the absence of clinical chorioamnionitis, abruptio placentae and fetal compromise. Prophylactic parenteral broad-spectrum antibiotics were given at admission. No treatment except antibiotics was initiated to delay delivery after 34 weeks. Management of PPROM women in the Czech Republic [14] differs substantially from most clinical guidelines. Management of PPROM is active except for PPROM pregnancies at <28 weeks of gestation, which are handled with expectant care. Timing for induction of labor or elective caesarean section depends on gestational age at membranes rupture (within 24 hours (h) in those with gestational age above 34.0 weeks, within 48 hours in those between 32.0 and 33.6 weeks of gestation, and within 72 hours after membrane rupture between 28.0 and 31.6 weeks). Fetal and maternal status were closely monitored until delivery. Maternal serum C-reactive protein (CRP) and white blood cell (WBC) concentrations were assayed upon admission and every subsequent day until delivery.

Ultrasound-guided transabdominal amniocentesis was performed at admission before the administration of corticosteroids, antibiotics or tocolytics. Cultures for aerobic and anaerobic bacteria, as well as polymerase chain reaction analysis (PCR) for genital mycoplasmas and *Chlamydia trachomatis*, were assayed immediately after collection. The results were available for clinical management. Protease inhibitors (Complete^TM^ Mini, EDTA-free Protease Inhibitor Cocktail; Roche Diagnostics, Basel, Switzerland) were added to the remaining amniotic fluid (40 µL per 1 mL of amniotic fluid). The amniotic fluid was centrifuged for 15 minutes at 2000x g to remove cells and debris, filtered (0.22-μm Syringe-driven filter; TPP, Trasadingen, Switzerland), divided into aliquots and stored at −70°C until analysis.

Twenty six proteins were tested in amniotic fluid using a multiple sandwich immunoassay based on flowmetric Luminex xMAP technology. Methodology used is described *in extenso* later in methods section.

MIAC was defined as a positive PCR for genital mycoplasmas and/or *Chlamydia trachomatis*, and/or a positive amniotic fluid culture. The presence of coagulase-negative *Staphylococcus epidermidis* in amniotic fluid was considered as a skin contaminant, except in women with high levels of amniotic fluid Interleukin (IL)-6 and IL-8. The amounts of genital mycoplasma DNA in amniotic fluid samples were also evaluated using real-time polymerase chain reaction. Specific technique used is also described later in methods section.

After delivery, the placenta was fixed in buffered formalin; tissue blocks from the placenta, umbilical cord and placental membranes were routinely embedded in paraffin and sections were stained with hematoxylin and eosin for a standard histological examination. Histopathological examinations were performed by a single pathologist who was blinded to the clinical status of the patients. The degree of polymorphonuclear leukocyte infiltration was evaluated separately according to criteria proposed by Salafia [15] in the free membranes (amnion and chorion-decidua), the chorionic plate and the umbilical cord. The diagnosis of HCA was determined based on grades 3–4 in the chorion-decidua, grades 3–4 in the chorionic plate, grades 1–4 in the amnion and/or grades 1–4 in the umbilical cord [15].

To evaluate the magnitude of intra-amniotic inflammatory response the data were analyzed using different proteins in amniotic fluid according to the presence of MIAC with HCA, the presence of HCA alone, the presence of MIAC alone, and a group with neither MIAC nor HCA (non-infection detected group).

### Amniotic fluid analyses: flowmetric Luminex xMAP technology

Amniotic fluid Interleukin (IL)-1β, IL-6, IL-8, IL-10, IL-12, IL-17, IL-18, soluble IL-6 receptor (sIL-6r), adiponectin, brain-derived neurotropic factor (BDNF), C-reactive protein (CRP), granulocyte macrophage colony stimulating factor (GM-CSF), insulin-like growth factor-binding protein (IGFBP)-1, IGFBP-3, interferon-(IFN)γ, leptin, monocyte chemotactic protein-1 (MCP-1), migration inhibiting factor (MIF), macrophage inflammatory protein-1 (MIP-1), matrix metalloproteinasis-9 (MMP-9), neutropin-3 (NT3), regulated on activation normal T-expressed and secreted (RANTES), tumor necrosis factor (TNF)-α, TNF-β, soluble TNF receptor-1 (sTNF-R1) and triggering receptor expressed on myeloid cells-1 (TREM-1) were analyzed at the Statens Serum Institute (Department of Clinical Biochemistry and Immunology, Copenhagen, Denmark) using a multiple sandwich immunoassay based on flowmetric Luminex xMAP technology in accordance with the workflow previously published [16]–[20]. The undiluted samples of amniotic fluid were measured in duplicate. Calibration curves were prepared in assay buffer (phosphate buffered saline containing 5 mL/L Tween 20 and 10 g/L bovine serum albumin). The means of intra-assay and inter-assay coefficients of variation were 6% and 12%, respectively. The defined working range described by Skogstrand *et al* [*19]*, *[20*] was used, due to the impossibility of obtaining cytokine-free amniotic fluid. This approach was considered to be a more accurate method of defining sensitivity than the commonly used signal-to-noise ratio (limit of detection). The detection level was defined as half of the lowest level in the working range in amniotic fluid (IL-1β 5 pg/mL, IL-6 19.5 pg/mL, IL-8 2.5 pg/mL, IL-10 10 pg/mL, IL-12 4 pg/mL, IL-17 4 pg/mL, IL-18 10 pg/mL, sIL-6r 19.5 pg/mL, Adiponectin 488.5 pg/mL, BDNF 10 pg/mL, CRP 200 pg/mL, GM-CSF 4 pg/mL, IGFBP-1 97.5 pg/mL, IGFBP-397.5 pg/mL, IFN-γ 4 pg/mL, leptin 97.5 pg/mL, MCP-1 2.5 pg/mL, MIF49 pg/mL, MIP-1 39 pg/mL, MMP-9 244 pg/mL, neutropin-3 (NT3) 39 pg/mL, RANTES 2.5 pg/mL, TNF-α 4 pg/mL, TNF-β 4 pg/mL, sTNF-R1 156.5 pg/mL, TREM-1 97.5 pg/mL). These analyses were performed by an investigator who was blinded to the clinical status of the women.

### Detection of genital mycoplasmas: The threshold cycle value of real-time PCR for genital mycoplasmas

The bacterial load of the genital mycoplasmas was assessed using the threshold cycle value (Ct value). DNA was isolated from the amniotic fluid with the QIAamp DNA Mini Kit (QIAGEN, Hilden, Germany) following the manufacturer's instruction. Real-time PCR was performed on a Rotor-Gene 6000 (QIAGEN, Hilden, Germany) with the commercial kit AmpliSens^®^ Ureaplasma/M. hominis-FRT (Federal State Institution of Science, Central Research Institute of Epidemiology, Moscow, Russia) to detect DNA of *Ureaplasma parvum, Ureaplasma urealyticum and Mycoplasma hominis* in a common PCR tube. Controls included internal control and PCR for beta-actin, a housekeeping gene, to examine the presence of inhibitors of the PCR reaction.

The Ct value is defined as the intersection between an amplification curve and a threshold line. This method measures the concentration of target DNA in the PCR reaction. The amount of target amplification increases at a rate of one log_10_ every 3.32 cycles. Thus, the higher the bacterial load of genital mycoplasmas in the amniotic fluid the lower the Ct values.

### Ethics

Written informed consent was obtained from all subjects. The Institutional Review Board approved the collection and use of these samples and information for research purposes (Ethics committee of University Hospital Hradec Kralove, Sokolska 581, 500 05, Hradec Kralove. March 19, 2008; No. 200804 SO1P).

### Statistical methods

Statistical analyses were performed using SPSS 19.0 for Windows XP OS (SPSS Inc., USA). Demographic and clinical characteristics were compared using the nonparametric Jonckheere-Terpstra test and Mann-Whitney *U* test and results are presented as medians (range). Categorical variables were compared using linear by linear association Chi-square and are presented as numbers (%). Spearman partial correlation was used to adjust data for gestational age at amniocentesis sampling. Differences were considered statistically significant at a confidence level of *p*<0.05 with two-sided alternative hypotheses.

## Results

### Demographic data

From July 2008 to October 2010, 174 women with a diagnosis of PPROM between 23.0 and 36.6 weeks of gestation were admitted to hospital. A total of 145 women met the inclusion criteria. However, amniotic fluid samples could not be retrieved from 30 women and the placenta was not sent for histopathological assessment in 8 women. Therefore, 107 were included in the final analysis. Gestational age at PPROM was less than 28.0 weeks in 18 (17%) women; between 28.0 and 31.6 weeks in 20 (19%) women; between 32.0 and 33.6 weeks in 25 (23%) women and beyond 34.0 weeks in 44 (41%) women. Median gestational ages at sampling and at delivery of the entire study population were 33.1 (range: 23.6–36.5) weeks and 33.2 (range: 24.0–36.5) weeks, respectively. Latency between membranes rupture and delivery was <96 h in 90% (96/107), <72 h in 78.5% (84/107) and <48 h in 63% (67/107) of all women. Despite active management in PPROM women, spontaneous delivery was observed in more than 70% of women ≥28.0 weeks. There were no differences in latency from PPROM to delivery among the different subgroups of population <28.0 weeks.

Maternal and neonatal characteristics of the entire study group are summarized in Table 1. The overall rate of MIAC was 44% (47/107) and HCA was found in 57% (61/107) of women. Considering presence or absence of MIAC and/or HCA, 31 women had MIAC and HCA, 30 women had only HCA, 16 women had only MIAC and in 30 women no infection was detected. Gestational age at PPROM was significantly earlier in the subgroup of women with MIAC and HCA. Since latency from PPROM to delivery, gestational age at delivery and birth weight may be influenced by the active management of PPROM pregnancies beyond 28 weeks of gestation in Czech Republic, data was adjusted by gestational age at amniocentesis sampling. Gestational age at delivery, latency from PPROM to delivery and birth weight remained significant lower in the subgroup with MIAC and HCA after adjustment.

**Table 1:** Maternal and neonatal characteristics in the different subgroups.

	MIAC + HCA N = 31	HCA alone N = 30	MIAC alone N = 16	Non-infection detected subgroup N = 30	*p*	p*
Maternal age	29 (18–40)	32 (25–44)	31.5 (24–44)	31 (19–40)	0.903	0.864
Nulliparity	12 (39)	18 (60)	9 (56)	20 (67)	0.190	0.055
Smoking	7 (22)	4 (13)	5 (31)	1 (3)	0.051	1.000
Gestational age at amniocentesis sampling (weeks)	30.5 (23.6–35.4)	33.3 (24–36.1)	34.1 (31.6–36.3)	34.2 (24.0–36.5)	<0.001	-
Latency from PPROM to amniocentesis (hours)	5 (2–16)	6 (1–22)	4 (1–23)	6.5 (1–16)	0.681	0.660
Latency from PPROM to delivery (hours)	34 (8–244)	39.5 (6–217)	16 (5–77)	19 (7–119)	0.019	0.016
Latency from PPROM to delivery in PPROM <28 weeks (hours)	64.5 (31–244)	118 (11–167)	-	63 (7–119)	0.880	0.419
CRP (mg/L)	9 (0–82)	9 (1–72)	5 (0–13)	5 (0–71.3)	0.014	0.016
WBC count at admission (x10^9^/L)	13 (4–26.8)	12 (6.6–20)	13.05 (7–19)	11 (7–24)	0.207	0.219
Gestational age at delivery (weeks)	31 (24.1–35.5)	33.5 (24.4–36.2)	34.1 (32.2–36.4)	34.2 (24.0–36.5)	<0.001	<0.001
Spontaneous delivery in PPROM ≥28.0 weeks	16/23 (69)	17/24 (71)	11/16 (69)	22/28 (78)	0.858	0.073
Birth weight (g)	1560 (470–2580)	2260 (495–3210)	2225 (1820–2910)	2270 (750–3460)	<0.001	<0.001
5- min Apgar score <7	2 (6)	4 (13)	0	1 (3)	0.264	0.364
Neonatal composite morbidity	13 (42)	10 (33)	5 (31)	7 (23)	0.556	0.127

MIAC: Microbial invasion of amniotic cavity. HCA: Histological chorioamnionitis. PPROM: Preterm prelabor rupture of membranes. CRP: C-reactive protein. WBC: White blood cells. Continuous variables were compared using a nonparametric Jonckheere-Terpstra test and presented as medians (range). Categorical variables were compared using linear-by-linear association Chi-square test and presented as numbers (%). Spearman partial correlation was performed to adjust by gestational at amniocentesis sampling (p*).

### Microbial invasion of amniotic cavity

The most common microorganism isolated from amniotic fluid was *Ureaplasma urealyticum* (n = 26). Other microorganisms isolated were: *Mycoplasma hominis* (n = 2), *Chlamydia trachomatis* (n = 3), *Streptococcus agalactiae* (n = 6), *Streptococcus* α *haemolyticus* (n = 2), *Streptococcus pneumoniae* (n = 1), coagulase-negative *Staphylococcus epidermidis* (n = 3), *Staphylococcus auricularis* (n = 1), *Staphylococcus hominis* (n = 1), *Enterococcus* spp (n = 1), *Enterococcus faecalis* (n = 1), *Haemophylus influenzae* (n = 1), *Fusobacterium* (n = 1), *Lactobacillus* spp (n = 3), *Bacillus spp* (n = 2), *Escherichia coli* (n = 1) and *Candida albicans* (n = 1). In eight cases, a polymicrobial infection in the amniotic fluid was detected. All isolations of coagulase-negative *Staphylococcus epidermidis* bacteria were considered as a positive amniotic fluid culture due their high expression of amniotic fluid IL-6 and IL-8.

In women with MIAC alone, the microorganisms isolated in amniotic fluid were: Ureaplasma urealyticum (n = 8) Mycoplasma hominis (n = 1), Streptococcus agalactiae (n = 2), Staphylococcus hominis (n = 1), Chlamydia trachomatis (n = 2), Candida albicans (n = 1), Staphylococcus auricularis (n = 1), Streptococcus pneumoniae (n = 1), Streptococcus α haemolyticus (n = 1, Bacillus spp (n = 1) and Lactobacillus spp (n = 1). Polymicrobial infection was present in five women.

### Intra-amniotic inflammatory response

Eight proteins (IL-1β, IL-12, IL-17, IL-18, IFN-γ, RANTES, TNF-α and TNF-β) had undetectable amniotic fluid levels in more than 50% of the samples and were excluded from further analyses. Therefore, eighteen proteins were analyzed to evaluate differences in the intra-amniotic inflammatory response.

A comparison of levels of amniotic fluid cytokines and neuropeptides in all subgroups is shown in Table 2. Of the 18 biomarkers evaluated, 14 presented significantly higher concentrations (measured by median (range)) in the subgroups with MIAC and HCA than in the others. On adjustment for gestational age at amniocentesis sampling, only the intra-amniotic inflammatory response mediated by IL-6, IL-10, BDNF, MCP-1, MIP-1 and MMP-9 remained significantly higher in the presence of both MIAC and HCA.

**Table 2:** Comparison of the levels of cytokines found in the different subgroups in PPROM.

	MIAC + HCA N = 31	HCA alone N = 30	MIAC alone N = 16	Non-infection detected subgroup N = 30	*p^1^*	*p^2^*	*p^3^*
IL-6	1576 (17–6659)	432 (28–3259)	198 (30–2672)	237 (40–1952)	<0.001	0.002	0.011
IL-8	456 (7–4000)	100 (4–4000)	24.5 (4–752)	34 (4–1262)	<0.001	0.434	0.097
IL-10	57 (4–2199)	16.5 (4–93)	12.5 (4–30)	14 (4–38)	<0.001	<0.001	0.246
sIL-6r	523 (86–2970)	239 (69–1176)	154 (65–1622)	227 (38–1843)	0.003	0.050	0.626
Adinopectin	72356 (22370–357223)	39467 (13571–376591)	30006 (14550–79970)	39690 (12044–22995)	0.004	0.431	0.842
BDNF	227 (10–930)	119.5 (10–481)	112 (10–250)	121 (10–308)	0.002	0.033	0.835
CRP	3357 (162–17786)	2572 (402–24601)	1619.5 (427–8921)	1806 (223–9965)	0.030	0.526	0.466
GMC-SF	37 (4–161)	13 (4–48)	11 (4–79)	18 (4–53)	0.010	0.050	0.429
IGFBP-1	32876 (6229–189996)	19017.5 (8294–159405)	19217 (8406–50332)	24212 (4781–172740)	0.599	0.799	0.609
IGFBP-3	32915 (17202–44264)	30950 (6316–41363)	25094.5 (11870–41700)	33237 (9359–43279)	0.200	0.591	0.557
Leptin	1097 (126–7672)	959 (64–5324)	349.5 (42–1994)	825 (137–6345)	0.117	0.738	0.629
MCP-1	802 (50–2500)	163.5 (37–1131)	91.5 (38–912)	130 (54–719)	<0.001	<0.001	0.532
MIF	8376 (1351–50000)	4521 (1768–50000)	4284.5 (1265–23173)	4909 (1313–21155)	0.045	0.686	0.680
MIP-1	412 (18–6355)	50.5 (9–666)	46 (4–377)	42 (4–165)	<0.001	<0.001	0.289
MMP-9	5323 (571–87534)	2291 (608–16325)	1872 (461–12247)	1872 (529–7951)	<0.001	0.007	0.196
NT3	398 (217–2368)	365.5 (120–1259)	370 (180–890)	410 (232–697)	0.440	0.190	0.673
sTNF-R1	1199 (168–7517)	479.5 (135–3779)	254.5 (158–2838)	352 (138–3943)	<0.001	0.111	0.203
TREM-1	227 (43–2790)	171.5 (58–598)	155.5 (50–673)	161 (60–579)	0.031	0.055	0.813

*p^1^*: Continuous variables were compared using a nonparametric Jonckheere-Terpstra test and presented as medians (range).

*p* ^2^: Spearman partial correlation was performed to adjust by gestational age at amniocentesis sampling.

*p^3^*: comparison between HCA alone, MIAC alone and non-infection detected subgroup.

Since current clinical management of pregnancies complicated with PPROM in most of countries is active beyond 34.0 weeks, inflammatory response was evaluated in women with PPROM <34.0 weeks and ≥34.0 weeks (Table 3 and 4).

**Table 3:** Comparison of the levels of cytokines found in the different subgroups in PPROM women <34 weeks.

	MIAC + HCA N = 26	HCA alone N = 17	MIAC alone N = 7	Non-infection detected subgroup N = 14	*p^1^*	*p^2^*
IL-6	2018.5 (17–6659)	568 (120–3259)	301 (62–2672)	284 (56–1952)	<0.001	0.054
IL-8	475.5 (7–4000)	110 (12–4000)	56 (8–752)	85.5 (4–1262)	0.001	0.199
IL-10	75 (4–2199)	21 (4–63)	13 (4–30)	19 (4–38)	<0.001	0.537
sIL-6r	694 (86–2970)	303 (69–1176)	231 (109–1622)	350 (69–1843)	0.201	0.692
Adinopectin	76449 (22370–357223)	66963 (18145–376591)	28285 (17489–79970)	68413.5 (24551–229905)	0.154	0.575
BDNF	268 (10–930)	191 (10–481)	132 (10–204)	131 (44–308)	0.027	0.382
CRP	3071 (162–17786)	2733 (402–20576)	3374 (691–8921)	1804.5 (223–8965)	0.307	0.486
GMC-SF	37.5 (4–161)	18 (4–33)	13 (4–79)	18.5 (4–32)	0.008	0.967
IGFBP-1	33689.5 (6229–172830)	27341 (15288–145255)	31254 (16833–50332)	42133 (7070–172740)	0.350	0.319
IGFBP-3	33651 (17202–44264)	33068 (7238–41363)	33742 (11870–41700)	33901 (17161–38206)	0.765	0.946
Leptin	1063 (126–6226)	1306 (64–5324)	355 (42–1342)	1124 (228–6345)	0.501	0.827
MCP-1	824 (50–2500)	171 (68–1131)	2972 (1759–12247)	2824.5 (626–7951)	0.002	0.913
MIF	8103 (1351–50000)	9025 (2832–50000)	7709 (4284–23173)	9041.5 (2266–21255)	0.883	0.712
MIP-1	495.5 (18–6355)	53 (11–176)	118 (53–912)	201.5 (62–719)	<0.001	0.672
MMP-9	7000 (571–87534)	3182 (1049–10076)	48 (12–377)	48 (17–165)	0.022	0.382
NT3	475 (235–2368)	481 (120–1259)	304 (180–644)	416 (277–692)	0.231	0.753
sTNF-R1	1219 (174–7517)	679 (309–3779)	328 (239–2838)	720.5 (215–3943)	0.066	0.539
TREM-1	228 (61–2790)	188 (58–598)	121 (67–351)	167.5 (60–457)	0.006	0.257

*p^1^* Continuous variables were compared using a nonparametric Jonckheere-Terpstra test and presented as medians (range).

*p^2^*: comparison between HCA alone, MIAC alone and non-infection detected subgroup.

**Table 4:** Comparison of the levels of cytokines found in the different subgroups in PPROM women ≥34.0 weeks.

	MIAC + HCA N = 5	HCA alone N = 13	MIAC alone N = 9	Non-infection detected subgroup N = 16	*p^1^*	*p^2^*
IL-6	1576 (378–3527)	401 (28–1323)	128 (30–1276)	174.5 (40–493)	0.003	0.168
IL-8	181 (171–1156)	58 (4–2411)	15 (4–77)	33 (4–233)	0.032	0.675
IL-10	19 (4–514)	14 (4–93)	12 (4–29)	10 (4–32)	0.154	0.331
sIL-6r	276 (129–846)	161 (90–808)	118 (65–463)	141.5 (38–562)	0.146	0.589
Adinopectin	68762 (25877–98918)	28011 (13571–189950)	31520 (14550–78013)	32832 (12044–57772)	0.257	0.818
BDNF	176 (74–360)	71 (10–154)	66 (10–250)	81.5 (10–271)	0.626	0.413
CRP	6555 (1888–17141)	2570 (685–246011)	1006 (427–3141)	1910.5 (618–9965)	0.183	0.675
GMC-SF	34 (4–99)	11 (4–48)	9 (4–27)	18 (4–5)	0.991	0.218
IGFBP-1	30859 (16670–189996)	1762 (8294–159405)	16140 (8406–49254)	21872.5 (4781–50354)	0.717	0.579
IGFBP-3	30889 (23371–35152)	20518 (6316–36763)	22700 (15358–29470)	2752.5 (9359–43279)	0.991	0.274
Leptin	1444 (281–7672)	355 (143–1513)	311 (138–1994)	23.5 (137–2905)	0.886	0.351
MCP-1	599 (303–2500)	161 (37–957)	60 (38–263)	109.5 (54–592)	0.020	0.552
MIF	11436 (3474–13335)	4053 (1768–15759)	03450 (1265–4615)	4173 (1313–15421)	0.333	0.746
MIP-1	102 (22–1460)	45 (9–666)	44 (4–124)	36 (4–102)	0.071	0.358
MMP-9	4772 (1510–6198)	1903 (608–16325)	1226 (461–2001)	1473 (529–5902)	0.097	0.797
NT3	262 (217–783)	350 (208–466)	541 (184–890)	372 (232–697)	0.235	0.433
sTNF-R1	425 (168–1714)	272 (135–1300)	231 (158–810)	258.5 (138–799)	0.202	0.607
TREM-1	119 (43–424)	133 (61–270)	158 (50–673)	179 (84–579)	0.403	0.351

*p^1^* Continuous variables were compared using a nonparametric Jonckheere-Terpstra test and presented as medians (range).

*p^2^*: comparison between HCA alone, MIAC alone and non-infection detected subgroup.

In PPROM <34.0 weeks, high intra-amniotic inflammatory response, mediated by IL-6, IL-8, IL-10, BDNF, GMC-SF, MCP-1, MIP-1, MMP-9 and TREM-1, was observed when both MIAC and HCA were present. In women with PPROM ≥34 weeks, fewer inflammatory biomarkers (IL-6, IL-8 and MCP-1) were involved compared to earlier gestational age. Finally, there were no differences in any of the amniotic fluid cytokines evaluated between women with MIAC or HCA alone and women without detected infection (Table 3, 4).

### Microorganism quantification in women with microbial invasion of the amniotic cavity

To further explore the relationship between women with MIAC and HCA and MIAC alone we used a quantitative bacterial approach. In women with a positive PCR for genital mycoplasma (19 women with MIAC and HCA and 9 women with MIAC alone), the amount of genital mycoplasma DNA was evaluated using real-time PCR. The presence of MIAC and HCA was associated with lower threshold cycle values (median 25.7, range, 13.1–31.6) compared to the group of MIAC alone (median 30.06, range, 26–35 *p* = 0.010). Therefore, the amount of genital mycoplasma DNA was significantly higher in women with MIAC and HCA than in those with MIAC alone.

Finally, no significant differences were observed in the inflammatory response between genital mycoplasma and other microorganisms isolated in women with MIAC alone (Table 5).

**Table 5:** Comparison of the levels of cytokines found in the women with MIAC alone according the microorganism isolated.

	Genital mycoplasma N = 6	Other microorganisms N = 10	*p*
IL-6	279 (61–2672)	198 (30–1276)	0.792
IL-8	24.5 (4–752)	28 (4–390)	0.958
IL-10	4 (4–26)	16 (4–30)	0.181
sIL-6r	181 (109–512)	128 (65–1622)	0.428
Adinopectin	27082 (17489–78013)	30875.5 (14550–79970)	0.492
BDNF	112 (10–158)	107.5 (10–250)	0.635
CRP	2119.5 (691–7263)	1232 (427–8921)	0.368
GMC-SF	4 (4–79)	13 (4–27)	0.428
IGFBP-1	36827 (11571–49254)	16971.5 (8406–50332)	0.118
IGFBP-3	25394 (11870–36848)	25094.5 (15358–41700)	0.792
Leptin	404.5 (42–1994)	311 (74–1473)	0.562
MCP-1	88.5 (38–899)	104 (47–912)	0.875
MIF	4725.5 (2119–20719)	3784 (1265–23173)	0.368
MIP-1	18 (12–377)	58 (4–127)	0.263
MMP-9	1893.5 (956–7496)	1750 (461–12247)	0.428
NT3	329 (180–683)	454.5 (184–890)	0.492
sTNF-R1	267 (239–863)	245.5 (158–2838)	0.368
TREM-1	124 (67–225)	158.5 (50–673)	0.368

Three women with polymicrobial invasion due to genital mycoplasmas and other microorganisms were considered in the subgroup of “other microorganisms”.

Continuous variables were compared using a nonparametric Mann-Whitney *U* test and presented as medians (range).

## Discussion

High intra-amniotic inflammatory response, mediated by IL-6, IL-10, BDNF, MCP-1, MIP-1 and MMP-9, was only identified in PPROM pregnancies with both MIAC and HCA. Therefore, the presence of not only MIAC but also HCA seems to be necessary to achieve high levels of inflammatory proteins in amniotic fluid.

The mechanism responsible for activation of the inflammatory pathway has been intensively studied. Intensity of intra-amniotic inflammatory response increases gradually when innate immunity defense is activated by the presence of bacteria in either the amniotic fluid or the fetal membranes. However, it has been suggested that intra-amniotic inflammation is elicited not only by the activation of pattern recognition receptors recognizing specific motifs on the surface of bacteria called pathogen-associated molecular pattern (PAMS) but also by endogenous molecules that signal tissue and cellular damage called “alarmins” [21]–[23]. Activated immunocompetent cells produce a broad spectrum of inflammatory mediators and chemokines responsible for attraction and migration of neutrophils, macrophages, and other immune cells to the placenta and fetal membranes.

Differences in intra-amniotic inflammatory response have previously been reported along the placenta [12] suggesting that the infiltration of neutrophils, which are the first line of defense, progressively advances from the chorio-decidua space through the amnion in response to infection. Moreover, the finding of fetal neutrophils into umbilical cord (funisitis) and in the chorionic plate vessels has been considered the last stage of inflammatory response associated with higher intra-amniotic inflammation and therefore a worse neonatal outcome [12], [24], [25]. Intra-amniotic inflammation has been extensively proposed as a marker of MIAC in preterm labor with or without rupture of membranes [6]–[9]. The chance in our study to examine the placental and umbilical cord compartment within a short time period of amniotic fluid sampling provides a unique opportunity to obtain a more complete assessment and a better knowledge of the inflammatory response in PPROM than previously reported. Accordingly, our data suggest firstly that the intra-amniotic inflammatory response, mediated by selected biomarkers, is identified only when both MIAC and HCA are present since MIAC or HCA alone are not associated with a higher inflammatory response when compared with a non-infection detected group. Secondly, most of the biomarkers involved in this inflammatory response are chemokines participating in the recruitment of cells from the second line of defense (MIP-1α for macrophages and MCP-1 for monocytes) and cytokines with anti-inflammatory (IL-10) or versatile (IL-6) effects. Surprisingly, no differences were found in IL-8, a strong attractant of neutrophils considered the first line of defense cells against microbial invasion. Finally, the number of biomarkers involved in this inflammatory response was higher when PPROM occurred before 34 weeks of gestation in line with previous data suggesting more intense inflammatory response at earlier gestational ages [1], [6], [9], [26], [27].

Altogether, our data suggest that intra-amniotic inflammatory response reflects a late stage of inflammation, where both placenta and amniotic cavity are involved. Whether MIAC or HCA alone subgroups represent a non-pathological colonization or an early stage of the infection that could be targeted by appropriate treatment, remains unanswered in this study. However, from a clinical point of view, MIAC or HCA alone do not seem to be able *per se* to activate the inflammatory pathway if the other counterpart is not affected. This finding is particularly relevant in the current clinical management of PPROM, where clinical decisions are mainly based on the diagnosis of MIAC and expectant management in very early pregnancies would be desirable.

To confirm our findings related to the low inflammatory response observed in women with MIAC alone, an analysis of the amount of genital mycoplasma DNA in amniotic fluid samples was performed in women with MIAC with or without HCA. A lower amount of genital mycoplasmas was observed in women with MIAC alone compared with those with both MIAC and HCA. These findings are in line previous research in which a lower intra-amniotic inflammatory response and a lower risk of subsequent development of HCA were observed in PPROM pregnancies with low bacterial load of genital mycoplasmas [28] Thus, in women with MIAC alone, no significant differences were observed in inflammatory response between genital mycoplasma detected by PCR and other microorganisms isolated by culture ratifying the fact that the lower inflammatory response observed in these women was not microorganism-dependent [28].

### Strengths and Limitations

One of the strengths of the study is the active management of PPROM in our cohort, with most women delivering within 96 h of membrane rupture (96/107). Since other studies with longer latency periods have previously reported the lack of relationship between HCA and latency period from membrane rupture to delivery [29], we hypothesize that placenta pathology at birth provided similar information as placenta pathology at amniocentesis sampling in most of our women.

Our study also presents some limitations. The study was performed in a single institution, which prevented the analysis of a larger sample size. Short and long term neonatal outcomes were not evaluated and therefore neonatal outcome regarding the presence or absence of inflammatory status was not evaluated. Also, we have not confirmed our microbial results with modern non-cultivation technologies. Consequently, the size of the subgroup with HCA alone and the non-infection detected subgroup might have been overestimated, while the subgroups with MIAC alone and with both MIAC and HCA might have been underestimated. However, in most clinical settings, these modern non-cultivation techniques are still not available, so clinical decisions have to be based on standard methods. Besides, a different non-infectious pathway to explain the activation of the intra-amniotic inflammatory response (as the activation by endogenous signals) has not been considered. Finally, other standard tests of microbial infection such as Gram staining, white blood count and glucose were not performed.

From a future perspective, assessment of inflammatory biomarkers should be taken into consideration in the clinical management of PPROM. Thus, the identification of a higher inflammatory response in non-invasive samples, such as from the cervix, seems to accurately reflect the status of the amniotic cavity [30]. Therefore, the clinical applicability of a rapid test to determine inflammatory response in amniotic fluid or non-invasive samples of pregnant women complicated with PPROM would probably improve the knowledge of intra-uterine status.

In summary, the identification of a high inflammatory status in amniotic fluid in women with PPROM seems to translate a worse infectious scenario in which MIAC and HCA are present. Therefore, information exclusively from amniotic fluid culture may be insufficient for clinical decision particularly in early gestational ages. These results support further research to establish the potential contribution of these inflammatory biomarkers in amniotic fluid and non-invasive samples to the clinical management of PPROM.
